# Effect of risedronate on bone loss at discontinuation of denosumab

**DOI:** 10.1016/j.bonr.2020.100290

**Published:** 2020-06-25

**Authors:** Michel Laroche, Guillaume Couture, Adeline Ruyssen-Witrand, Arnaud Constantin, Yannick Degboé

**Affiliations:** a Centre de Rhumatologie [Rheumatology Center], CHU Purpan, 1 place du Dr. Baylac, TSA 40031, 31059 Toulouse cedex 9, France; b Université Toulouse III - Paul Sabatier, 118 Route de Narbonne, 31062 Toulouse cedex 9, France

**Keywords:** Osteoporosis, Bone mineral density, Risedronate, Denosumab

## Abstract

**Purpose:**

The occurrence of multiple vertebral fractures was reported after denosumab discontinuation. The use of bisphosphonates following denosumab has been suggested to prevent this bone loss. The aim of our observational trial was to evaluate the ability of risedronate to prevent the bone loss related to denosumab discontinuation in post-menopausal osteoporosis.

**Methods:**

Eighteen female patients, aged 69.8 years (56–79), were followed. All patients were prescribed 35 mg of risedronate per week for 3 months, starting when the next denosumab injection would have been administered. We measured BMD at denosumab initiation (T0), denosumab withdrawal (T1), and nine months after the discontinuation of risedronate (1 year post-denosumab: T2).

**Results:**

1 year after denosumab discontinuation, the mean bone loss at the spine was – 4.6 ± 5.2% for the total population, −0.3 ± 2.3% in patients with prior exposure to bisphosphonates, −6.3 ± 5.7% in patients with prior exposure to teriparatide, and − 7.6 ± 3.5% in naïve patients. Spine BMD loss after the risedronate bridging therapy (T2 vs. T1) was significantly lower in patients who experienced prior exposure to bisphosphonates, when compared to naïve patients (p = .0190) and to patients with prior teriparatide exposure (p = .0176). 1 year after denosumab discontinuation, the mean densitometric loss at the hip was −1.8 ± 3.4% in the total cohort, −0.6 ± 1.8% in the patients previously treated with bisphosphonates, −1.5 ± 4.7% in the patients previously treated with teriparatide, and − 4.2 ± 0.6 in naïve patients. The mean densitometric loss during the off-denosumab period was lower in patients with previous bisphosphonate exposure than in naïve patients (p = .043) and in patients with previous exposure to teriparatide (p = .05).

**Conclusions:**

Three months of risedronate treatment does not prevent bone loss in patients who have not been treated with bisphosphonates before denosumab.

## Highlights

• Three months of risedronate
• Ineffectiveness
• Bone loss
• discontinuation of denosumab

## 1 Introduction

Multiple vertebral fracture has recently been reported after denosumab discontinuation (Anastasilakis et al., 2017; Anastasilakis et al., 2019). The off-treatment extension studies with this drug showed a significant rebound in bone turnover markers, exceeding the initial levels for 48 months (Bone et al., 2011a, Bone et al., 2011b). This intense bone turnover was paralleled with a loss in bone mineral density (BMD) from the first year following denosumab discontinuation, both at spine and hip sites (Popp et al., 2018).

Discontinuation strategies for osteoporosis treatments have been proposed. According to the recommendations of the GRIO (French Osteoporosis Research and Information Association), treatment discontinuation after the first drug course could be considered in patients achieving all of the following conditions: no fracture during treatment, no new risk factors for osteoporosis, no significant decrease in spine or hip BMD. A femoral hip T-score ≥ −2.5 has been proposed in patients with a history of severe fracture (Briot et al., 2018). This consensus of experts has been proposed for bisphosphonates. However, given the absence of carry-over effect with denosumab and the rebound in bone turnover after discontinuation, the safety of a similar strategy after denosumab is not assured. The work of the European Calcified Tissue Society (ECTS) suggested a post-denosumab (dmab) bisphosphonate therapy to prevent/reduce rebound in bone turnover (Tsourdi et al., 2017). This strategy remains to be validated. The aim of our study was to evaluate the ability of a 3-month risedronate bridging therapy to reduce BMD loss after denosumab discontinuation.

## 2 Patients - methods

### 2.1 Patients

In our Rheumatology Center, we proposed to 18 women, treated for at least 2 years with denosumab, who met the aforementioned criteria for treatment discontinuation (no new fracture, no comorbidity that contributes to falls or fractures, T-score above − 2.5 on the three sites) (N = 16) or who presented side effects to this treatment (N = 2: One interruption was due to cataract, and one was due to requirements for dental implants) to switch from dmab to risedronate.

### 2.2 Treatment regimen

The patients started risedronate (RIS) treatment six months after the last denosumab injection. The risedronate regimen was one 35 mg tablet per week for 3 months. The patients were informed of the precautions associated with risedronate administration, especially the intake with non-mineral water half an hour before breakfast.

Taking treatment according to these precautions was verified during follow-up consultations.

All patients were given a vitamin D supplement (oral vitamin D3 100,000 units, every two months). Calcium intake was optimised to 1 g per day, with oral supplementation if needed. This protocol was approved by the ethical committeee of Toulouse II and patients provided informed consent.

### 2.3 Outcome measures

Baseline (T0) was defined as the initiation of denosumab. We collected biological and densitometric data at dmab discontinuation (T1) and one year later (T2; after the 3-month risedronate course and then 9 months off-treatment). Common biochemical blood tests including calcium, phosphorus, 25OH-vitamin D and parathyroid hormone were carried out at T1 and T2. Serum collagen type 1 cross-linked C-telopeptide (CTX) levels were measured at T1 and T2, with IDS-iSYS immunoassay system using chemiluminescent detection (normal values: 350–650 pg/ml for menopausal women). All biological parameters were collected in fasting patients and in real time.

BMD was measured at spine and hip sites using dual X-ray absorptiometry (Lunar Prodigy, GE Healthcare® UK) by a single experimenter. Vertebral fracture assessment (VFA) was systematically assessed at T0, T1 and T2. BMD variations are described as change from baseline (T0). CV = 1% for the spine, 1.2% for the hip.

### 2.4 Statistical analysis

DXA variables were described as means and SD or medians when the distribution was not normal. We analyzed patients with prior exposure to bisphosphonates (BP), teriparatide (TPTD), and naïve patients.

The comparison of the groups between T0, T1 and T2 was carried out using the ANOVA test. The comparison between T1 and T2 using a *t*-test for Gaussian-distributed variables, and a Wilcoxon-Mann-Whitney test for non-Gaussian-distributed variables. Correlation between denosumab duration and BMD variation was assessed by Pearson correlation calculations. A p-value <.05 was considered as statistically significant with a 95% confidence interval. All analyses were performed using the GraphPad Prism v5.00 (GraphPad Software, Inc., La Jolla, CA 92037 USA).

## 3 Results

Eighteen female patients, mean age 70.2 ± 6.9 years, were analyzed. Ten patients had vertebral fractures, 6 had non-vertebral fractures and 3 had osteoporosis without fractures with a T-score below −3 SD at the initiation of denosumab.

Patients' data are reported in Table 1. Six patients benefited from a prior bisphosphonate treatment before denosumab. Three patients had zoledronate and 3 had alendronate. The mean bisphosphonate treatment duration was four years. Eight patients have been treated with teriparatide (18 months) before denosumab: among these 8 patients, 5 had had no treatment before teriparatide, 2 had been treated with risedronate, one with alendronate. 4 patients had no prior anti-osteoporosis treatment before denosumab.

**Table 1:** Demographic, biological and densitometric characteristics.

Patient	Age	% Spine variation T0-T1	% Spine variation T0-T2	% Hip variation T0-T1	% Hip variation T0-T2	DMAB duration (months)	Prior treatment	Post-RIS Crosslaps (pg/ml)
M.	75	+8.5	−2.5	+3.4	+0.1	36	0	244
F.	56	+4.4	−5.6	+6.7	+1.7	36	teriparatide	269
R.	62	+11.0	+10.0	+6.0	+4.0	48	zoledronate	120
D.	73	+0.5	−3.0	0.0	−4.5	48	0	60
L.	73	+15.0	+15.0	+3.0	+3.0	48	alendronate	361
Le.	69	+8.0	+10	+1.0	−1.0	24	alendronate	200
G.	76	+2.5	+1.5	+0.0	+1.5	24	zoledronate	NA
C.	75	+12.0	+3.0	–	–	60	teriparatide	730
Re.	74	+4.5	−0.5	+3.4	0.0	36	teriparatide	482
Ma.	70	+12.0	+2.0	+4.2	−0.1	36	0	68
G.	56	+15.0	+10.8	+3.5	−1.9	36	teriparatide	320
La.	78	+5.0	+7.0	+5.0	+2.6	48	alendronate	150
Ca.	74	+1.0	+3.0	0.0	0.0	12	teriparatide	84
Cal.	79	+12.0	+8.0	+3.5	+5.0	48	alendronate	NA
Mar.	72	+5.5	+3.5	+3.2	+8.7	36	teriparatide	Nd
E.	62	+23.0	+17.5	+5.5	+9.5	24	teriparatide	420
K.	70	+3.6	−2.4	+3.5	−1.0	48	0	283
Mb.	69	+16.0	−1.0	+13.0	+7.0	48	teriparatide	760
Mean (SD)	70.2 (6.9)	+8.9 (6.1)	+4.2 (6.5)	+3.8 (3.1)	+2.0 (3.8)	38.7 (12.0)	–	303.4 (60–760)
Naïve	72.0 (2.4)	+6.2 (5.1)	−1.5 (2.3)	+2.8 (1.9)	−1.4 (2.1)	42.0 (6.9)	–	163.8 (60–283)
Prior BP	72.8 (6.4)	+8.9 (4.7)	+8.6 (4.4)	+3.1 (2.3)	+2.5 (2.1)	40 (12.4)	–	207.7 (120–361)
Prior TPTD	67.3 (8.1)	+10.2 (7.5)	+3.8 (7.3)	+5.0 (4.1)	+3.6 (4.7)	36 (14.3)	–	437.9 (84–760)
P value (naïve vs. BP)	0.6429	0.5143	0.0190	0.8810	0.0429	>0.9999	–	0.6571
P value (naïve vs. TPTD)	0.4768	0.3010	0.0990	0.5545	0.05	0.4081	–	0.0424
P value (BP vs. TPTD)	0.1658	0.8282	0.2138	0.4231	0.9132	0.5275	–	0.1576

ALN: alendronate, BP: bisphosphonates, CTX: crosslaps, DMAB: denosumab, NA: not available, TPTD: teriparatide, RIS: risedronate, SD: standard deviation, ZOL: zoledronate.

T0 = start DMAB; T1: switch denosumab-risedronate; T2 = one year after stopping DMAB.

The median T-score at the end of denosumab was −2.3 at the spine (−3; −1.5) and − 2.2 at the hip (−2.8; −2).

The 2 patients whose T-score was less than − 2.5 were the patients who had to stop denosumab because of side effects and which we included all the same in the study.

The mean treatment duration with denosumab was 38.7 months (SD = 12), ranging from 12 months (discontinuation of treatment due to cataract) to 48 months.

**Fig. 1:**
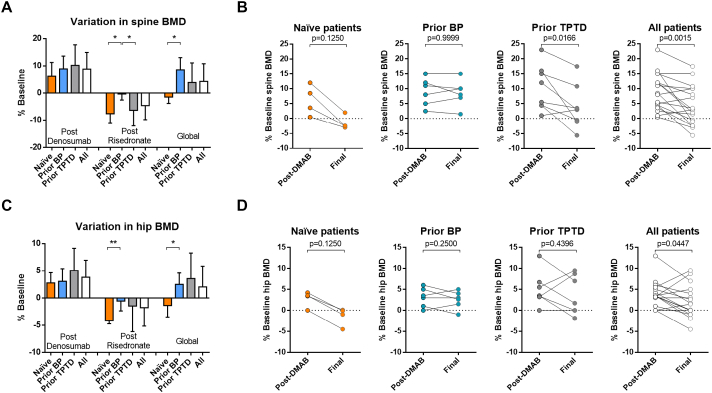
Variation in spine and hip bone mineral density. BMD: bone mineral density, BP: bisphosphonates, Global: after denosumab + risedronate sequence, TPTD: teriparatide. Spine and hip bone mineral density were assessed at denosumab withdrawal (post-denosumab) and at the end of the 1-year off-denosumab period (post-risedronate and global).

BMD variations are reported in Table 1 and Fig. 1.

The mean densitometric gain from baseline (T1 vs. T0) in the spine with denosumab was +8.9 ± 6.1% for the total population. It was +8.9 ± 4.7% in patients with prior exposure to bisphosphonate, +10.2 ± 7.5% in patients with prior exposure to TPTD, and + 6.2 ± 5.1% in naïve patients (Fig. 1A). The mean densitometric gain after denosumab was not statistically different across groups p = .51; between naïve vs. BP patients: p = .3; between naïve vs. TPT patients: p = .30; between BP vs. TPT patients: p = .82 (Fig. 1A).

We then assessed the evolution of spine BMD, nine months after the 3-month course of risedronate (T2 vs. T1) (Table 1, Fig. 1A-B). 1 year after denosumab discontinuation, the mean bone loss at the spine was −4.6 ± 5.2% for the total population, −0.3 ± 2.3% in patients with prior exposure to bisphosphonates, −6.3 ± 5.7% in patients with prior exposure to teriparatide, and − 7.6 ± 3.5% in naïve patients. Spine BMD loss after the risedronate bridging therapy (T2 vs. T1) was significantly lower in patients who experienced prior exposure to bisphosphonates, when compared to naïve patients (p = .0190) and to patients with prior teriparatide (p = .0176) (Fig. 1A). Thus, 1 year after denosumab discontinuation, the mean densitometric change from baseline (T2 vs. T0) in the spine was +4.2 ± 6.5% for the total population, +8.6 ± 4.4% in patients with prior exposure to bisphosphonates, +3.8 ± 7.3% in patients with prior exposure to teriparatide, and − 1.5 ± 2.3% in naïve patients. The mean densitometric gain was lower in naïve patients than in patients with prior exposure to BP (p = .04). No statistical difference between the others groups. We found no statistical correlation between the duration of denosumab exposure and the final spine BMD variation (Pearson r = −0.1107, p = .6618) and no correlation between the duration of denosumab exposure and the loss of BMD after risedronate treatment (Pearson: r = −0.15, p = .35).

At denosumab withdrawal, the mean densitometric gain from baseline at the hip (T1 vs. T0) was +3.8% (SD 3.1) in the total population, +3.1% (SD 2.3) in patients previously treated with bisphosphonates, +5.0% (SD 4.1) in patients with prior exposure to teriparatide, and + 2.8% (1.9) in naïve patients (Fig. 1C). These mean densitometric gains were not statistically different across groups (Fig. 1C). 1 year after denosumab discontinuation (T2 vs. T1), the mean densitometric loss at the hip was −1.8 ± 3.4% in the total cohort, −0.6 ± 1.8% in the patients previously treated with bisphosphonates, −1.5 ± 4.7% in the patients treated previously with teriparatide, and − 4.2 ± 0.6 in naïve patients. The mean densitometric loss during the off-denosumab period was lower in patients with previous bisphosphonate exposure than in naïve patients (p = .043, Fig. 1C) and in patients with previous exposure to teriparatide (p = .05). No significant difference was observed between the other groups.

One year after denosumab discontinuation, the mean densitometric change from baseline (T2 vs. T0) at the hip was +2.0% (SD 3.8) in the total cohort, +2.5% (SD 2.1) in the patients previously treated with bisphosphonates, +3.6% (SD 4.7) in the patients treated previously with teriparatide, and − 1.4% (SD 2.1) in naïve patients (Table 1 and Fig. 1C-D). The mean densitometric change was lower in naïve patients than in patients with prior exposure to BP (p = .05). There was no statistical difference between the others groups. We found no statistical correlation between the duration of denosumab exposure and the final hip BMD variation (Pearson r = 0.0359, p = .8912) or the BMD loss after risedronate (Pearson: r = −0.28, p = .1).

All the patients had normal levels of calcium, phosphorus, vitamin D and PTH at denosumab withdrawal (T1) and end of the study (T2).

CTX levels at the discontinuation of denosumab were < 33 pg/ml (detection limit) in all patients. At the end of the study, they were 303 pg/ml (60–760) in the total population, 208 (120–361) in patients previously treated with bisphosphonates, 438 pg/ml (84–760) in patients previously treated with teriparatide, and 164 pg/ml (60–263) in the naïve patients. CTX levels were significantly higher in the patients with prior exposure to teripatide when compared to the naïve patients (p = .024). There was non-correlation between post-risedronate CTX levels and BMD changes post-denosumab (p: 0.35).

Three months after the discontinuation of risedronate, one patient had a new vertebral fracture.

This patient had 2 vertebral fractures at the start of treatment with denosumab and had lost 7% of BMD between T1 and T2.

No other serious adverse event was observed during the study.

## 4 Discussion

In our study, we analyzed the effect of a 3-month risedronate bridging therapy after denosumab discontinuation, on BMD in post-menopausal women, with regards to their prior treatment.

This treatment partially decreases bone loss when denosumab is stopped in patients not previously treated with bisphosphonates.

We chose to administer risedronate for 3 months because we thought this strategy was more relevant than the realization of a zoledronate infusion, whose infusion time is currently under study.

These studies must evaluate the most opportune moment to carry out the infusion: at the time when the injection of dmab should be carried out or 3 or 6 months later, at the maximum increase in CTX (6).

We chose a little persistent action bisphosphonate for a short time because we thought that it would be sufficient to block the hyper-resorptive rebound caused by the denosumab stoppage.

The main limitations of our study were the small number of patients, the lack of statistical power for sub-group analyses, and potentially the short duration of post-denosumab risedronate regimen. However, given the lack of available randomized controlled trials, here we provide data to help the clinicians to optimise the strategy for denosumab discontinuation.

Denosumab increased spine and hip BMD in most patients. In accordance with previous reports, a part of this gain has been lost in the 1-year off-denosumab period (Popp et al., 2018). Despite risedronate bridging therapy, naïve patients lost the benefit in BMD acquired with denosumab, with levels inferior to baseline levels in some patients. The extent of bone loss after risedronate was higher in the spine than in the hip. The anti-resorbing activity and carry-over effects of risedronate are inferior to those of alendronate and zoledronate. These properties could explain its inefficiency to prevent spine bone loss post-denosumab discontinuation. Moreover, the use of risedronate for a short period of time could be an additional explanation. Despite this bone loss, biological assessment of bone resorption (CTX), was within normal limits for post-menopausal women 12 months after the discontinuation of denosumab and 9 months after the discontinuation of risedronate.

What about the other bisphosphonates? In a cohort of 6 women with post-menopausal osteoporosis from the FREEDOM trial, Reid et al. showed that a single infusion of zoledronate significantly reduced spine (but not hip) bone loss associated with the discontinuation of 7 years of continuous denosumab treatment: During denosumab treatment, bone mineral density (BMD) in the spine increased 18.5% (P = .006), and total hip BMD by 6.9% (P = .03). Post-zoledronate BMDs were measured 18–23 months after treatment, and there were significant declines at each site (P_spine_ = 0.043, P_hip_ = 0.005). Spine BMD remained significantly above the pre-denosumab baseline (+9.3%, P = .003), but hip BMD was not significantly different from the baseline (−2.9%).

BMD assessment performed 18–23 months after zoledronate treatment indicated a final spine gain of +9.3% from the pre-denosumab baseline and the absence of a significant final modification in the hip (Reid et al., 2017). Thus, a single infusion of zoledronate was ineffective to prevent hip bone loss after denosumab.

The same authors reported the effects of different strategies for romosozumab/denosumab discontinuation in 19 women involved in the FRAME trial (Horne et al., 2018). Four patients had no post-trial treatment, 11 had zoledronate (given within a median delay of 65 days post-trial) and 5 had risedronate. The BMD assessment performed one year after bisphosphonates introduction showed there was no significant loss of BMD from trial-end to 1 year in all treatment groups, except in hip BMD for patients taking risedronate. In the DAPS study, Freemantle et al. showed that a 1-year course of alendronate following a 1-year denosumab treatment prevented spine and hip bone loss (Freemantle et al., 2012). However, it is important to point out that this study (randomized, open-label, crossover) was designed to compare adherence to denosumab versus alendronate in 250 patients, and not to primarily focus on the BMD outcome. It is possible that the beneficial effect of zoledronate and alendronate in these last two trials is due to the fact that the patients only received one year of treatment with denosumab before the switch.

Bone loss related to the discontinuation of denosumab was shown to increase with the duration of treatment (Tsourdi et al., 2017). The study by McClung et al. indicates a preventive effect of the bisphosphonates on bone loss when denosumab is discontinued, but the number of patients followed was low and the treatments prescribed too heterogeneous to define for any strategy (McClung et al., 2017).

We showed that patients with a prior exposure to bisphosphonates had almost no bone loss. This finding was observed at the spine and hip. We don't know if they would have evolved in the same way without risedronate.

Patients from the FREEDOM study were excluded if they used intraveinous bisphosphonates or more than 3 years of oral bisphosphonates. However, no point is made about the patients reported by Reid et al. (Reid et al., 2017). Only 3 out of 19 patients from the FRAME study reported by Horne et al. were previously exposed to bisphosphonates (alendronate, maximum of 4 months) (Horne et al., 2018).

In our study, patients who had prior treatment with bisphosphonates over an extensive period (an average of four years) had lower bone loss as well as a lower increase in CTX. Our finding is in line with the study of Uebelhart et al. which shows that CTX remains low with the discontinuation of denosumab in 14 out of 17 patients who received prior treatment with bisphosphonates: CTX remained in the pre-menopausal range in 14 out of 17 patients who discontinued denosumab after multiple injections (4.1 ± 1.4, range 2–7) but were previously exposed to BPs (mean exposure 6.9 ± 5.8 years). In contrast, in 12 patients who discontinued denosumab after multiple injections (5, 3–9) without prior exposure to BPs, mean CTX levels as measured on average 11.3 months (6–23) after the last dmab injection were above the upper limit of premenopausal range (mean + 114%, 28–320%, p = .003–0.005 vs. previous BPs.

Moreover, these authors showed that CTX does not increase with the discontinuation of denosumab in patients who had only one injection, which confirms the fact that the resulting hyper-resorption and bone loss increase with the length of treatment (Uebelhart et al., 2017).

The 2 most informative studies seem to us to be that of Anastasilakis, recently published (11), and that of Lehmann and Aeberli (2017) (12), whose results are contradictory:

Lehman report 22 cases of women with post-menopausal osteoporosis under the care of a resident rheumatologist between May 2010 and March 2017. Patients, either previously treated with BP (n = 13) or without BPs (n = 9), were started on subcutaneous denosumab (60 mg every 6 months). All women received five injections and were then given a single dose of zoledronic acid 6 months after the fifth injection of denosumab.

The 9 patients who had not been treated with bisphonates before dmab, had a gain of BMD in the spine under dmab of + 7.4% and a loss of − 3.1% despite zoledronate. At the hip, the gain was 3.1% and the loss was − 2.3%.

Anastasilakis et al. recently investigated the efficacy of zoledronate to prevent this bone loss in a randomized multicenter study including 57 women with post-menopausal osteoporosis who were treated with denosumab (mean duration 2.2 years) and discontinued treatment after achieving osteopenia. Women were randomized to receive a single 5 mg infusion of zoledronate (ZOL) (n = 27) six months after the last dmab injection or two additional 60 mg injections of denosumab (dmab) (n = 30). At 24 months lumbar spine–bone mineral density (LS-BMD) was not different from the baseline in the zol group, but decreased in the dmab group by 4.82% ± 0.7% (p < .001) from the 12-month value; the difference in BMD changes between the two groups and the primary endpoint of the study was statistically significant (p = .025). Results of femoral neck (FN)-BMD changes were similar. These strategies therefore seem effective, moreover, they show here that the variability of BMD changes following zol treatment is not related to the rate of bone turnover at the time of the infusion or at any other time point assessed during treatment. Therefore, the authors conclude that there is no reason to delay the administration of zol beyond 6 months after the last dmab injection.

Would the zoledronate infusion have been as effective in patients, like ours, treated twice as long (4 years versus 2 years) by denosumab before the switch?

In our study, in patients treated with teriparatide before denosumab, bone loss was twice as low as in naïve patients. At the end of the denosumab-risedronate sequence, the overall result is positive with an overall gain in bone mass of +3.8% at the spine and + 3.6% at the hip. What would have happened without risedronate? Further studies are needed.

One patient had a new vertebral fracture shortly after risedronate withdrawal. No fracture cascade was noted but 16/18 patients had a T-score above − 2.5 when denosumab was discontinued and no recent fractures. Interestingly, alendronate following denosumab did not prevent a fracture cascade in two patients, reported by Lamy et al. (Lamy et al., 2019a).

The literature currently lacks precise data to define the best strategy for denosumab discontinuation in order to prevent bone loss and possibly fractures. For elderly patients with a high risk of fracture, based on the 10-year studies, a “lifelong” treatment could be envisioned (Laroche, 2016). However, the risk of atypical femoral fractures and osteonecrosis of the jaw should be taken into account. In younger patients who benefited from teriparatide/denosumab or romososumab/denosumab treatment sequences, resulting in major densitometric gains, and who have not benefited from the protective role of prior bisphosphonates, the best strategy remains to be defined (Lamy et al., 2019b). The same goes for the few patients who have experienced adverse effects with denosumab.

## 5 Conclusion

Our study shows that risedronate does not prevent bone loss in patients who had not been treated with bisphosphonates before denosumab. The duration of treatment chosen was perhaps too short given the low anti-resorptive power of this bisphosphonate and the low persistence of this bisphosphonate.

## CRediT authorship contribution statement

**Michel Laroche:** Conceptualization, Writing - original draft, Writing - review & editing. **Guillaume Couture:** Data curation. **Adeline Ruyssen-Witrand:** Methodology. **Arnaud Constantin:** Writing - review & editing. **Yannick Degboé:** Writing - review & editing.

## Declaration of competing interest

Michel Laroche, Guillaume Couture, Adeline Ruyssen-Witrand, Arnaud Constantin and Yannick Degboe declare that they have no conflict of interest about this work.
